# Assessment of Cardiovascular Risk in Long-Term Type 1 Diabetes: The Role of Adiponectin and Leptin

**DOI:** 10.3390/medicina62061037

**Published:** 2026-05-27

**Authors:** Gergana Chausheva, Sevim Shefket, Yana Bocheva, Kaloyan Tsochev, Tatiana Chalakova, Natalya Usheva, Yoto Yotov, Violeta Iotova

**Affiliations:** 1 Department of Clinical Laboratory, Medical University-Varna, 9002 Varna, Bulgaria; sevim.shefket@mu-varna.bg (S.S.); yana.bocheva@mu-varna.bg (Y.B.); 2 Department of Pediatrics, Medical University-Varna, 9002 Varna, Bulgaria; kaloyan.tsochev@mu-varna.bg (K.T.); iotova_viol@abv.bg (V.I.); 3 1st Department of Internal Diseases, Medical University-Varna, 9002 Varna, Bulgaria; tatyana.chalakova@mu-varna.bg (T.C.); yoto.yotov@mu-varna.bg (Y.Y.); 4 Department of Social Medicine and Health Care, Medical University-Varna, 9002 Varna, Bulgaria; nataly_usheva@mu-varna.bg

**Keywords:** long-standing type 1 diabetes (T1D), cardiovascular risk (CVR), adiponectin (ADNC), leptin (LEP), Steno Type 1 Risk Engine (ST1RE), ESC 2019 guidelines

## Abstract

*Background and Objectives*: Individuals with long-standing type 1 diabetes (T1D) remain at elevated cardiovascular risk (CVR). Adiponectin (ADNC) and leptin (LEP) are adipokines involved in metabolic and vascular homeostasis, yet their relevance for CVR stratification in T1D is unclear. This study examined the associations of ADNC and LEP with CVR categories derived from the Steno Type 1 Risk Engine (ST1RE) and the 2019 ESC Guidelines on Diabetes, Pre-diabetes and Cardiovascular Diseases (ESC 2019), with particular attention to potential sex-related differences. *Materials and Methods*: A cross-sectional study included 124 adults with long-standing T1D and 59 age- and sex-matched non-diabetic participants. Serum ADNC and LEP concentrations were measured using standardized immunoassays. CVR was assessed using ST1RE and ESC 2019 algorithms. Multivariable models adjusted for age and body mass index (BMI) were used to examine determinants of adipokine concentrations and their associations with CVR categories. *Results*: Participants with T1D exhibited higher circulating ADNC concentrations than non-diabetic individuals (*p* < 0.001). LEP levels were significantly associated with sex (*p* < 0.001). In adjusted analyses, sex and BMI remained significantly associated with adipokine concentrations. Higher LEP levels showed a significant positive association with higher ESC 2019 CVR category (OR 1.84; 95% CI 1.19–2.84; *p* = 0.006), whereas ADNC and the LEP × sex interaction showed no significant association. Neither adipokine was significantly associated with ST1RE-derived risk categories. *Conclusions*: In adults with long-standing T1D, LEP showed an association with ESC 2019-based CVR stratification, whereas neither LEP nor ADNC was associated with ST1RE-derived risk categories.

## 1. Introduction

Type 1 diabetes (T1D) is a chronic autoimmune condition characterized by the destruction of pancreatic β-cells and lifelong insulin dependence. Despite substantial improvements in insulin therapy and metabolic monitoring, cardiovascular disease (CVD) remains the major cause of premature mortality among individuals with T1D [1,2,3]. Epidemiological studies show that the risk of cardiovascular events remains significantly elevated compared with the general population, even in patients with apparently adequate glycemic control, suggesting that traditional cardiometabolic markers may not fully capture disease burden [3,4].

Adipose tissue has high endocrine activity secreting a broad range of bioactive mediators—adipokines, which exert effects on vascular tone, insulin signaling, immune responses, and atherogenesis [5,6]. Among these, adiponectin (ADNC) and leptin (LEP) are two of the most intensively studied [5]. Their significance in T2D and obesity is well established [6]; however, their roles in T1D are less clearly delineated [1].

ADNC is an adipokine with insulin-sensitizing, anti-inflammatory, and anti-atherogenic properties [7]. Paradoxically, contrary to its reduced levels in obesity and T2D [8], circulating ADNC is often elevated in T1D, particularly in patients with long disease duration and diabetic nephropathy—a phenomenon referred to as the “adiponectin paradox” [8]. Possible explanations for this phenomenon include loss of pancreatic β-cells, tissue resistance to adiponectin, and reduced renal clearance [6,7,8]. Additional explanations are the compensatory response to an underlying chronic inflammation, reduced kidney function, and impaired clearance or catabolic age-related breakdown processes [9,10,11]. Although increased levels have been associated with favorable metabolic traits, several studies suggest that elevated ADNC may reflect counter-regulatory responses and may not necessarily convey cardioprotective effects [11,12]. Whether elevated ADNC represents a compensatory mechanism or contributes to pathological vascular remodeling in T1D remains unclear.

LEP, another major adipokine, is primarily involved in appetite regulation, energy expenditure, and immune activation. Hyperleptinemia has been linked to vascular inflammation, oxidative stress, endothelial dysfunction, and platelet activation [13,14]. More recent data suggest that LEP may contribute to early vascular calcification and plaque instability [15,16]. While LEP concentrations are strongly influenced by adiposity, studies in T1D indicate considerable heterogeneity, with elevated levels observed particularly in patients with long-standing disease and intensive insulin therapy [17]. Importantly, LEP may exhibit sexually dimorphic effects, contributing more strongly to CVD progression in men than in women [18].

Both ADNC and LEP exhibit pronounced sex-dependent differences, influenced by hormonal regulation and body fat distribution. Circulating LEP levels are consistently higher in women than in men, even after adjustment for BMI, which has been attributed to differences in fat distribution and the regulatory effects of sex steroids [19,20,21]. Similarly, ADNC levels tend to be higher in women, with evidence suggesting modulation by hormonal factors and metabolic characteristics rather than adiposity alone [22,23]. Importantly, both adipokines are closely linked to adiposity, and adjustment for BMI and sex is essential when interpreting their biological variability and potential clinical relevance.

CVR estimation in T1D remains suboptimal. Most validated risk algorithms, including SCORE or contemporary European risk charts, were originally developed in non-diabetic or T2D populations and tend to underestimate CVD risk in T1D [24,25]. The 2019 ESC Guidelines on Diabetes, Pre-diabetes and Cardiovascular Diseases (ESC 2019), developed in collaboration with the EASD, provide a structured framework for CVR stratification in diabetes, incorporating factors such as diabetes duration, albuminuria, chronic kidney disease, hypertension, and target-organ damage [24]. However, these guidelines rely primarily on clinical parameters and do not include molecular biomarkers that may reflect early endothelial dysfunction. The Steno Type 1 Risk Engine (ST1RE) was developed to address these limitations by incorporating diabetes-specific predictors [26], yet its performance is considered complementary rather than definitive, underscoring the need for novel biomarkers.

The aim of this study was to evaluate the associations of serum ADNC and LEP with estimated CVR in adults with long-standing T1D, using ST1RE and ESC 2019, with additional emphasis on potential sex-specific differences.

## 2. Materials and Methods

### 2.1. Study Design

A cross-sectional study with prospective recruitment was conducted at the University Hospital “St. Marina” (Varna, Bulgaria), which serves as a tertiary referral center for diabetes care in the region, between 2018 and 2020.

### 2.2. Participants

Patients with T1D were consecutively recruited during routine outpatient visits at the hospital’s endocrinology clinic.

#### 2.2.1. Inclusion Criteria

Diagnosed T1D for >15 years; age ≥ 18 years; stable clinical condition at the time of enrollment and written informed consent.

#### 2.2.2. Exclusion Criteria

Other autoimmune or systemic inflammatory diseases; severe cognitive or physical disability; acute metabolic decompensation within the preceding 3 months (e.g., ketoacidosis or severe hypoglycemia); pregnancy; recent major cardiovascular events (defined as myocardial infarction, ischemic or hemorrhagic stroke, transient ischemic attack, coronary or peripheral revascularization, hospitalization for unstable angina, or acute decompensated heart failure within the preceding 6 months); severe chronic diabetic complications (vision-threatening retinopathy or advanced nephropathy); and participation in another clinical trial. Advanced nephropathy was defined as severe renal impairment and/or clinically significant diabetic kidney disease likely to substantially affect metabolic and inflammatory status.

The non-diabetic group was recruited from the general population through public announcements and voluntary participation. Eligibility required absence of diabetes and major chronic diseases. The comparison group was frequency-matched to the T1D cohort by age, sex, and BMI.

A total of 183 adults were enrolled, including 124 individuals with long-standing T1D and 59 metabolically healthy non-diabetic participants serving as a comparison group.

### 2.3. Study Size

The primary objective of the study was exploratory; no formal a priori sample size calculation was performed.

### 2.4. Data Collection

All data were collected at study enrollment using standardized clinical examination, laboratory testing, and structured interviews.

### 2.5. Socio-Demographic Variables

The following socio-demographic variables were recorded:

- Age (years)
- Sex (male/female)
- Duration of diabetes (years; T1D group only)

Smoking status was assessed by structured interview and categorized as current smoker (yes/no). Former smokers were classified as non-smokers if smoking cessation had occurred ≥12 months prior to enrollment.

Physical activity was self-reported using a structured questionnaire and categorized as vigorous, moderate, walking ≥10 min consecutively, or sedentary behavior. Data were collected for the preceding 7 days, including both the frequency (days) and duration (hours and minutes) of activity. Vigorous physical activity was defined as activity requiring substantial physical effort leading to shortness of breath (e.g., fast cycling, weight lifting, digging, aerobic exercise). Moderate physical activity was defined as activity requiring moderate effort causing slight shortness of breath (e.g., carrying light loads, cycling at a regular pace, doubles tennis). Walking included movement during work and at home, transportation, and any walking performed for recreation, exercise, or leisure. Sedentary behavior included time spent sitting at work, at home, or during leisure activities (e.g., watching television).

### 2.6. Anthropometric Measurements

- Body weight (kg)
- Height (m)
- Body mass index (BMI), calculated as weight (kg)/height^2^ (m^2^)

### 2.7. Clinical Parameters

- Systolic blood pressure (SBP) was measured in the seated position after 5 min of rest using an automated sphygmomanometer. Two consecutive measurements were obtained, and their average was used for analysis, in accordance with routine clinical practice.
- History of CVD was determined based on documented medical records and included previous myocardial infarction, stroke, transient ischemic attack, or peripheral arterial disease occurring more than 6 months prior to enrollment.

### 2.8. Laboratory Measurements

Blood and urine samples were collected under standardized conditions. Blood for HbA1C analysis was drawn into K2EDTA tubes. Serum was separated by centrifugation at 2500× *g* for 15 min and stored at −70 °C until batch analysis. First morning urine samples were collected in the amount of 20 mL, centrifuged for 15 min at 2500 *g* and used for albuminuria determination. Serum was obtained in a vacutainer with a gel separator and centrifuged for 15 min at 2500 *g* (6–8 mL). Serum was used for creatinine (µmol/L), ADNC (µg/mL), and LEP (ng/mL) testing.

HbA1c (%) was measured using a DCCT/NGSP-standardized immuno-inhibition assay and expressed as the percentage of total hemoglobin. LDL-cholesterol (mmol/L) was calculated using the Friedewald formula. Serum creatinine (µmol/L) was measured by colorimetric determination of the creatinine–picrate complex in an alkaline medium without deproteinization using a kinetic assay. Analyses were performed on an ADVIA Chemistry 1800 system (Siemens). Albuminuria (mg/L) was measured by immuno-turbidimetric analysis on Olympus AU600.

Estimated glomerular filtration rate (eGFR) was calculated using the CKD-EPI equation and expressed as mL/min/1.73 m^2^.

Serum ADNC and LEP concentrations were measured using commercial ELISA kits. According to manufacturer specifications, the analytical characteristics were as follows:

ADNC: Human Adiponectin ELISA (BioVendor, Brno, Czech Republic)

- Lower limit of quantification (LLOQ): 0.6 µg/mL
- Limit of detection (LOD): 0.026 µg/mL
- Analytical range: 1–100 µg/mL
- Intra-assay coefficient of variation (CV): <5%
- Inter-assay CV: <7%
- Reference ranges for males: BMI (kg/m^2^) < 25: 10.9 ± 4 μg/mL; 25–30: 8.8 ± 4 μg/mL; >30—23 ± 2.8 μg/mL; for females BMI (kg/m^2^) < 25: 13.6 ± 5.4 μg/mL; 25–30: 13.9 ± 8.6 μg/mL; >30: 11.4 ± 3.8 μg/mL.
- Cross–reactivity: none with LEP, resistin, LEPR

LEP: Leptin ELISA Kit (DIA Source, Mont-Saint-Guibert, Belgium)

- Lower limit of quantification (LLOQ): 0.2 ng/mL
- LOD: 0.04 ng/mL
- Analytical range: 0.5–60 ng/mL
- Intra-assay CV: <13.3%
- Inter-assay CV: <12.7%
- Reference ranges for males: BMI (kg/m^2^) 18–24: 0.5–3.2 ng/mL; 25–29: 0.5–14.6; 30–56: 2.5–42.1 ng/mL; for females: BMI (kg/m^2^) 14–18: 0.5–0.7 ng/mL; 18–24: 0.5–7.9 ng/mL; 25–29: 4.1–14.5; 30–56: 5.5–40.4 ng/mL.
- Cross reactivity: none with IL-1α; IL-1β; IL-4, IL-6, IL-8, IL-10; IL-15, TNF-α; TNF-β; IFN-γ; IGF-1; insulin; glucagon.

All assays were performed in duplicate; values outside analytical range were remeasured after appropriate dilution. No sample exhibited concentrations below the LLOQ.

### 2.9. CVR Assessment

#### 2.9.1. Steno Type 1 Risk Engine (ST1RE)

The ST1RE model estimates 10-year risk of combined fatal and non-fatal cardiovascular events (ischemic heart disease, stroke, peripheral arterial disease). The model incorporates: age, sex, and duration of diabetes, previous CVD, systolic blood pressure, albuminuria, HbA1c, estimated glomerular filtration rate (eGFR), LDL-cholesterol, smoking status, and physical activity. The ST1RE calculator is available online at [26].

Risk categories were defined as: low (<10%), moderate (10–20%), and high (≥20%).

#### 2.9.2. The 2019 ESC Guidelines on Diabetes, Pre-Diabetes and Cardiovascular Diseases (ESC 2019)

The guidelines provide a structured framework for 10-year CVR estimation in individuals with diabetes integrating clinical risk factors, target-organ damage, and established cardiovascular disease [24]. Patients with T1D were classified into three groups based on guideline criteria [24]:

- Moderate risk—adults with short diabetes duration (<10 years) and no additional risk factors.
- High risk—T1D duration ≥10 years or coexistence of at least one major CVR factor, such as hypertension, dyslipidemia, smoking, obesity, or family history of premature CVD.
- Very high risk—presence of target-organ damage (e.g., albuminuria, reduced eGFR, retinopathy), three or more major risk factors, established atherosclerotic cardiovascular disease (e.g., myocardial infarction, stroke/TIA, PAD), or early-onset T1D with long disease duration (>20 years).

Although ESC 2019 allows classification into moderate, high, and very high risk, the moderate-risk category was not represented in this cohort because all participants had diabetes duration >15 years. Therefore, ESC 2019 risk was analyzed as a binary variable (high vs. very high).

### 2.10. Statistical Analysis

Statistical analyses were performed using SPSS software, version 19.0 (IBM Corp., Armonk, NY, USA). Continuous variables are presented as median with interquartile range (IQR), and categorical variables as number (percentage). Differences between T1D and non-diabetic groups were assessed using the Mann–Whitney U test for continuous variables. Comparisons among three or more groups were performed using the Kruskal–Wallis test. Categorical variables were compared using the χ^2^ test or Fisher’s exact test when appropriate. To examine determinants of adipokine concentrations and formally test sex effects while accounting for adiposity, general linear models (GLMs) were fitted with log-transformed ADNC and LEP as dependent variables and with group, sex, age, BMI, and the group × sex interaction as predictors.

To assess the association between circulating adipokines and cardiovascular risk categories derived from ST1RE and ESC 2019, proportional-odds ordinal logistic regression models were applied. The proportional odds assumption was evaluated using the test of parallel lines. Effect modification by sex was examined by including an interaction term between the log-transformed adipokine concentration and sex (log[adipokine] × sex) to assess whether the association between adipokines and CVR categories differed by sex. ST1RE categories (low, moderate, high) were analyzed using proportional-odds ordinal logistic regression due to their ordered nature. ESC 2019 risk classification was analyzed as a binary outcome (high vs. very high), as no participants were classified as moderate risk in the present cohort. This approach, while necessary, reduces category granularity and may limit statistical sensitivity.

A complete-case analysis was performed. No substantial missing data were present for the variables included in the analysis.

All tests were two-tailed, and statistical significance was defined as *p* < 0.05.

## 3. Results

### 3.1. Study Population

A total of 183 individuals were included: 124 participants with long-standing T1D and 59 non-diabetic individuals. Baseline demographic, clinical, and laboratory characteristics are presented in Table 1. The two groups were comparable with respect to age and BMI. As expected, HbA1c levels were significantly higher in the T1D group (*p* < 0.001). HbA1c levels did not differ significantly between males and females with T1D (*p* > 0.05), suggesting that glycemic control was unlikely to confound sex-related differences in adipokine concentrations. Systolic blood pressure was modestly higher in T1D participants (*p* = 0.005). LDL-cholesterol, eGFR, smoking status, and physical activity did not differ significantly between groups.

### 3.2. Serum Adipokine Concentrations

ADNC levels were significantly higher in individuals with T1D than in the non-diabetic group (*p* < 0.001). In contrast, serum LEP concentrations did not differ between groups (*p* = 0.933) (Table 1).

### 3.3. Sex-Specific Differences in Adipokine Levels: Multivariable Analysis

Multivariable GLM models were fitted with log-transformed adipokine concentrations as dependent variables, adjusting for age and BMI, to formally assess sex-related differences (Table 2). For LEP, sex was independently associated with circulating levels (β = 1.15, 95% CI 0.83–1.47, *p* < 0.001). BMI was the strongest independent determinant (β = 0.126, *p* < 0.001), and age demonstrated a modest positive association (β = 0.011, *p* = 0.023). No significant group × sex interaction was observed (*p* = 0.639). For ADNC, sex remained independently associated with higher concentrations (β = 0.49, 95% CI 0.20–0.79, *p* = 0.001). BMI was inversely associated with ADNC (β = –0.042, *p* < 0.001). No significant group × sex interaction was detected (*p* = 0.585). The adjusted effect of group (T1D vs. non-diabetic group) did not reach statistical significance (*p* = 0.097).

### 3.4. Associations Between Circulating Adipokines and Cardiovascular Risk Categories

To evaluate the relationship between circulating adipokines and CVR stratification, proportional-odds ordinal logistic regression models were applied. ST1RE risk categories (low, moderate, high) and ESC 2019 risk categories (high, very high) were modeled as ordinal dependent variables. ADNC and LEP concentrations were log-transformed prior to analysis. Because both ST1RE and ESC 2019 algorithms incorporate established clinical risk factors (e.g., age, blood pressure, lipid parameters, diabetes duration, albuminuria, and renal function), no additional covariates were included in the models to avoid over-adjustment. This approach was intended to isolate the association between adipokine levels and algorithm-derived CVR categories without re-adjusting for variables already embedded within the respective risk models. Accordingly, odds ratios (ORs) reflect the relationship between adipokine levels and algorithm-derived CVR categories.

In proportional-odds ordinal regression models (Table 3), neither LEP nor ADNC was significantly associated with ST1RE-derived CVR categories. For ESC 2019 classification, higher log-transformed LEP levels were significantly associated with increased odds of belonging to a higher risk category (OR 1.84; 95% CI 1.19–2.84; *p* = 0.006), whereas ADNC was not associated with ESC-defined risk. Importantly, these findings reflect an association with risk classification and should not be interpreted as evidence of predictive ability for future cardiovascular events.

No significant interaction between LEP and sex was observed (*p* = 0.392).

Regression models were applied with ST1RE (low/moderate/high) and ESC 2019 (high/very high) CVR categories as dependent variables. Panel A presents proportional-odds ordinal logistic regression, whereas Panels B and C present binary logistic regression models. Adipokine concentrations were log-transformed prior to analysis. Each biomarker (LEP and ADNC) was evaluated in separate univariable models in Panels A and B. Panel C presents a model including LEP, sex, and their interaction term. ORs represent the change in odds of belonging to a higher risk category per 1-unit increase in log-transformed biomarker concentration. No additional covariates were included to avoid over-adjustment, as clinical variables are embedded within the respective risk algorithms. The proportional odds assumption was not violated for the ST1RE model (*p* > 0.05).

## 4. Discussion

This study examined the associations of LEP and ADNC with CVR categories derived from ST1RE and the 2019 ESC Guidelines in adults with long-standing T1D. Several principal observations emerged.

First, both adipokines demonstrated pronounced sex-related differences that persisted after adjustment for BMI and age. Second, only LEP was significantly associated with ESC 2019 risk categories. Third, neither adipokine showed a statistically significant association with ST1RE-derived CVR categories. Importantly, formal interaction testing did not demonstrate significant sex modification of the association between LEP and ESC-defined CVR.

Together, these findings suggest that circulating LEP may reflect aspects of CVR burden captured by the ESC 2019 classification, whereas ADNC appears less informative in this context.

### 4.1. Sex-Specific Differences in Adipokines

Sex-related patterns were evident for both adipokines in the present cohort. Earlier reports have shown that such differences do not necessarily appear in childhood or adolescence. For instance, Kaza et al. (2022) reported comparable ADNC concentrations between male and female adolescents with T1D, aged (mean ± SD) 14.8 ± 3.4 years [27]. Lausten-Thomsen et al. (2015) observed no sex-based variation in a large cohort of healthy non-obese Danish students aged 6–18 years [22]. Divergence in ADNC levels has been suggested to emerge gradually with maturation, potentially influenced by changes in androgen exposure during puberty, with the disparity becoming more pronounced in adulthood [28].

In population-based studies of adults, such as the analysis by Kuo et al. (2011) involving more than 4800 participants, sex-related differences in ADNC were attributed primarily to metabolic alterations rather than to variations in fat mass or BMI [23]. In our cohort, individuals with long-standing T1D exhibited higher ADNC levels compared with non-diabetic participants, consistent with previous findings and meta-analytic evidence demonstrating elevated adiponectin concentrations in T1D populations [27,29].

For LEP, numerous studies have documented higher circulating levels in females than in males, commonly attributed to differences in fat distribution and to the regulatory effects of sex steroids [19,20,21]. Experimental work has shown that adipocytes derived from female donors secrete greater amounts of LEP under standardized culture conditions, and estradiol enhances LEP release in adipose tissue explants from women but not from men [21]. Clinical observations parallel these findings: LEP concentrations decline after menopause, suggesting estradiol-dependent modulation, although not all studies identify a direct effect of estrogen administration on circulating LEP [19,20,21]. Conversely, androgens appear to exert inhibitory effects; testosterone levels in men correlate inversely with serum LEP independently of BMI, and in vitro exposure to testosterone suppresses LEP production in human adipocytes [20].

In multivariable GLM analyses, sex remained independently associated with both LEP and ADNC after adjustment for age and BMI, while no significant group × sex interaction was observed.

### 4.2. LEP and CVR Classification

A central finding of this study is the significant association between circulating LEP concentrations and ESC 2019 CVR categories. Higher log-transformed LEP levels were associated with increased odds of belonging to the very high ESC risk category. Although LEP is closely related to adiposity and reflects energy balance, our findings suggest that its association with ESC-defined CVR is not fully explained by BMI alone. This indicates that LEP may reflect additional pathophysiological processes beyond central obesity, including low-grade inflammation, endothelial dysfunction, and vascular remodeling [30,31,32,33]. In contrast, ADNC, despite its established metabolic role, did not demonstrate a similar association, which may reflect differences in biological function or the presence of compensatory mechanisms in long-standing T1D [12].

In contrast, LEP was not significantly associated with ST1RE-derived risk categories. This divergence may reflect conceptual and structural differences between the two algorithms. ESC 2019 classification emphasizes the presence of target-organ damage, cumulative risk factor burden, and established atherosclerotic disease, potentially capturing pathophysiological processes more closely linked to inflammatory and vascular pathways influenced by LEP [24]. In addition, ESC 2019 classification is partly driven by categorical clustering of conventional risk factors and target-organ damage, which may amplify associations with biomarkers reflecting systemic processes such as inflammation and vascular dysfunction. ST1RE, by contrast, integrates continuous clinical variables within a multivariable risk equation specifically calibrated for T1D populations, which may attenuate the relative contribution of any single biomarker [26]. Additionally, ESC 2019 classification in the present cohort was analyzed as a binary outcome due to the absence of moderate-risk participants. This reduced category granularity may have influenced the observed associations by limiting variability across risk groups.

LEP is increasingly recognized as a contributor to vascular dysfunction through pro-inflammatory, pro-atherogenic, and sympatho-excitatory mechanisms. Experimental and clinical data link hyperleptinemia to endothelial dysfunction, oxidative stress, arterial stiffness, and atherosclerosis [30,31,32,33]. LEP levels increase following initiation of insulin therapy in T1D and are often higher in patients receiving intensive insulin regimens [27,34], which may contribute to sustained elevations in long-standing disease.

Associations between LEP and structural vascular changes have also been reported. Positive relationships with carotid intima–media thickness have been observed in children and adolescents with T1D [35] and in adult populations [36]. Meta-analytic evidence confirms correlations between higher LEP levels and increased arterial rigidity and coronary calcification progression [36], as well as adverse cardiac remodeling in coronary artery disease [6].

Our findings extend these observations by demonstrating that circulating LEP is associated with guideline-based ESC CVR stratification in adults with long-standing T1D. Importantly, these findings should be interpreted as associations with risk classification rather than evidence of predictive ability for future cardiovascular events, given the cross-sectional design.

Importantly, although baseline LEP concentrations differ substantially by sex, formal interaction testing did not reveal statistically significant sex modification of the LEP–ESC association. Thus, while absolute levels are sex-dependent, the strength of association with ESC-defined CVR appears comparable in males and females within this cohort.

### 4.3. ADNC: Discrepancies and Possible Explanations

Despite its traditionally protective metabolic profile, ADNC did not demonstrate significant associations with either ST1RE or ESC 2019 risk categories. This finding is consistent with the so-called “adiponectin paradox,” observed in chronic disease states in which elevated ADNC may reflect compensatory upregulation in response to inflammation, renal dysfunction, endothelial injury, or catabolic stress rather than cardioprotection [12,37].

In T1D, persistent hyperadiponectinemia is well described and may reflect long-term exposure to exogenous insulin, chronic microvascular stress, and altered lipid metabolism [27,37]. Under these conditions, circulating ADNC concentrations may lose discriminatory capacity with respect to CVR classification. The absence of association in both models supports this interpretation.

### 4.4. Clinical Implications

The present findings suggest that circulating LEP may reflect CVR burden as captured by ESC 2019 classification in adults with long-standing T1D. However, in the absence of longitudinal cardiovascular outcomes, LEP should be interpreted as a biomarker associated with algorithm-derived risk categories rather than as a predictor of incident cardiovascular events.

The lack of association between adipokines and ST1RE categories further indicates that biomarker relevance may depend on the structural properties and conceptual framework of the applied risk model. These findings should be interpreted with caution, given the cross-sectional design and moderate sample size, which limit conclusions regarding predictive performance. Accordingly, adipokines should be considered as biomarkers associated with CVR categories rather than as predictors of future cardiovascular events.

Further longitudinal studies are required to determine whether LEP has independent prognostic value in individuals with long-standing T1D.

### 4.5. Limitations

Several limitations warrant consideration. The cross-sectional design precludes causal inference. The absence of longitudinal cardiovascular outcome data limits interpretation to risk classification rather than event prediction. The moderate sample size may have reduced power for detecting subtle interaction effects. Finally, ESC 2019 classification was operationalized as a two-level variable due to the long diabetes duration of the cohort, precluding evaluation of moderate-risk categories. Nevertheless, strengths of this study include the inclusion of adults with long-standing T1D, standardized biochemical assessment, and an analytical approach that examined sex-specific associations and modeled risk categories.

Residual confounding cannot be excluded. Factors such as adiposity, insulin dose and treatment intensity, renal function, and systemic inflammation may influence adipokine levels and could contribute to the observed associations, despite the rationale for not including additional covariates in the regression models.

The exclusion of participants with advanced nephropathy may have reduced variability in renal-related CVR markers, which should be considered when interpreting the findings.

The present study is based on data and analyses that form part of the doctoral dissertation of G. Chausheva, defended at the Medical University of Varna “Prof. Dr. Paraskev Stoyanov”, Bulgaria, in 2023, entitled “Laboratory Cardiovascular Risk Assessment in Individuals with Long-Standing Type 1 Diabetes Mellitus—Adipokines, Osteoprotegerin, Asymmetric Dimethyl-Arginine” [38]. The thesis was archived in the institutional open-access repository; however, it has not been previously published in a peer-reviewed scientific journal. This study is based on a previously characterized cohort; however, the present analysis focuses on distinct biomarkers (adiponectin and leptin) and addresses different pathophysiological mechanisms and research questions.

## 5. Conclusions

In adults with long-standing T1D, a higher ESC 2019 CVR category was associated with higher LEP levels, but not with ADNC. Neither adipokine was significantly associated with ST1RE-derived risk categories. Sex-related differences in adipokine concentrations were evident, but no significant interaction between LEP and sex was observed for ESC-defined risk. These findings support further investigation of LEP as a biomarker reflecting cardiovascular risk burden in T1D, particularly in longitudinal outcome-based studies. However, given the cross-sectional design and moderate sample size, the observed relationships should be interpreted as associations rather than causal or predictive.

## Figures and Tables

**Table 1:** Baseline characteristics of the study groups.

Variable	Non-Diabetic Group	T1D Group	*p*-Value
Age (years)	47.0 [41.5–52.0]	43.0 [36.0–50.0]	0.057
Sex (Male/Female)	33/26	66/58	0.853
BMI (kg/m^2^)	26.01 [22.62–30.76]	24.79 [22.67–27.97]	0.210
Systolic BP (mmHg)	121.13 [105.87–136.39]	128.17 [109.3–147.04]	**0.005**
HbA1c (%)	5.30 [5.10–5.65]	8.18 [7.33–9.51]	**<0.001**
LDL-cholesterol (mmol/L)	3.12 [2.28–3.96]	3.25 [2.4–4.1]	0.133
eGFR (mL/min/1.73 m^2^)	101.76 [92.25–109.54]	104.05 [85.39–111.72]	0.622
Albuminuria (mg/L)	5.25 [5.00–8.98]	10.10 [5.00–32.38]	**<0.001**
Current smoking, *n* (%)	28 (47.5%)	54 (43.5%)	0.623
Physical activity (past 7 days):	—	—	—
Vigorous, *n* (%)	34 (57.6%)	61 (49.2%)	0.286
Moderate, *n* (%)	34 (57.6%)	80 (64.5%)	0.369
Walking ≥10 min consecutively, *n* (%)	58 (98.3%)	118 (95.2%)	0.300
Sedentary, *n* (%)	50 (84.7%)	104 (83.9%)	0.800
Prior CVD/history of CVD	0 (0)	0 (0)	—
Duration of diabetes (years)	—	24.0 [19.0–29.2]	—
ADNC (µg/mL)	7.62 [5.79–11.76]	12.24 [8.19–18.54]	**<0.001**
LEP (ng/mL)	4.09 [2.26–6.20]	3.83 [1.43–7.44]	0.933

Notes: Continuous data are presented as median (IQR), and categorical data are reported as absolute and percentage values. Mann–Whitney U test used for continuous variables; χ^2^ for categorical. Physical activity data represent the proportion of participants reporting engagement in each activity category over the preceding 7 days. Categories are not mutually exclusive; therefore, percentages do not sum to 100% within each group. significant values (*p* < 0.05) are indicated in bold.

**Table 2:** Multivariable GLM analysis of determinants of circulating adipokine concentrations.

Outcome	Variable	β	95% CI	*p*-Value
**Log (LEP)**	Group:			
	Non-diabetic	Reference		
	T1D	−0.074	−0.676–0.527	0.808
	Sex:			
	Male	Reference		
	Female	1.151	0.830–1.472	**<0.001**
	Group × Sex (T1D × female)	0.092	−0.294–0.477	0.639
	Age	0.011	0.002–0.020	**0.023**
	BMI	0.126	0.105–0.147	**<0.001**
**Log (ADNC)**	Group:			
	Non-diabetic	Reference		
	T1D	0.466	−0.085–1.017	0.097
	Sex:			
	Male	Reference		
	Female	0.495	0.201–0.789	**0.001**
	Group × Sex (T1D × female)	−0.098	−0.451–0.255	0.585
	Age	0.004	−0.005–0.012	0.375
	BMI	−0.042	−0.061–−0.022	**<0.001**

Notes: Significant values (*p* < 0.05) are in bold.

**Table 3:** Association between circulating adipokines and CVR categories.

**Panel** ** A. ST1RE Risk Category (Low/Moderate/High): ordinal logistic regression**			
**Predictor**	**OR**	**95% CI**	***p*** **-Value**
log (LEP)	1.36	0.96–1.93	0.086
log (ADNC)	0.92	0.53–1.59	0.774
**Panel B. ESC 2019 risk category (high/very high): binary logistic regression**			
**Predictor**	**OR**	**95% CI**	***p*** **-Value**
log (LEP)	1.84	1.19–2.84	**0.006**
log (ADNC)	0.79	0.39–1.58	0.502
**Panel C. ESC 2019 risk category (high/very high) model: binary logistic regression including sex interaction (LEP)**			
**Predictor**	**OR**	**95% CI**	***p*** **-Value**
log (LEP)	2.88	1.30–6.36	**0.009**
Sex (female vs. male)	0.66	0.13–3.40	0.619
log (LEP) × sex (female)	0.66	0.21–2.07	0.482

## Data Availability

The data supporting this study are available from the corresponding author upon reasonable request.

## Abbreviations

The following abbreviations are used in this manuscript:

T1D	Type 1 diabetes
T2D	Type 2 diabetes
ADNC	Adiponectin
LEP	Leptin
CVR	Cardiovascular risk
ST1RE	Steno Type 1 Risk Engine
ESC 2019	2019 ESC Guidelines on Diabetes, Pre-diabetes and Cardiovascular Diseases
BMI	Body mass index
CVD	Cardiovascular disease
OR	Odds ratio
GLM	General linear models
eGFR	Estimated glomerular filtration rate

## References

[1] Rawshani A., Rawshani A., Franzén S., Eliasson B., Svensson A.M., Miftaraj M., McGuire D.K., Sattar N., Rosengren A., Gudbjörnsdottir S. (2017). Mortality and Cardiovascular Disease in Type 1 and Type 2 Diabetes. N. Engl. J. Med..

[2] Haji M., Erqou S., Fonarow G.C., Echouffo-Tcheugui J.B. (2023). Type 1 diabetes and risk of heart failure: A systematic review and meta-analysis. Diabetes Res. Clin. Pract..

[3] Julián M.T., Pérez-Montes de Oca A., Julve J., Alonso N. (2024). The double burden: Type 1 diabetes and heart failure-a comprehensive review. Cardiovasc. Diabetol..

[4] Livingstone S.J., Levin D., Looker H.C., Lindsay R.S., Wild S.H., Joss N., Leese G., Leslie P., McCrimmon R.J., Metcalfe W. (2015). Scottish Diabetes Research Network epidemiology group; Scottish Renal Registry. Estimated life expectancy in a Scottish cohort with type 1 diabetes, 2008–2010. JAMA.

[5] Fasshauer M., Blüher M. (2015). Adipokines in health and disease. Trends Pharmacol. Sci..

[6] Zhao S., Kusminski C.M., Scherer P.E. (2021). Adiponectin, Leptin and Cardiovascular Disorders. Circ. Res..

[7] Shklyaev S., Melnichenko A., Volevodz N., Falaleeva N., Ivanov S., Kaprin A., Mokrysheva N. (2021). Adiponectin: A pleiotropic hormone with multifaceted roles. Probl. Endokrinol..

[8] Menzaghi C., Trischitta V. (2018). The Adiponectin Paradox for All-Cause and Cardiovascular Mortality. Diabetes.

[9] Sowka A., Dobrzyn P. (2021). Role of Perivascular Adipose Tissue-Derived Adiponectin in Vascular Homeostasis. Cells.

[10] Walowski C., Herpich C., Enderle J., Braun W., Both M., Hasler M., Müller M., Norman K., Bosy-Westphal A. (2023). Analysis of the adiponectin paradox in healthy older people. J. Cachexia Sarcopenia Muscle.

[11] Kalkman H.O. (2021). An Explanation for the Adiponectin Paradox. Pharmaceuticals.

[12] Vankova D., Kiselova-Kaneva Y., Ivanova D. (2023). New insights of the adiponectin paradox. Scr. Sci. Ca Pharm..

[13] Pérez-Pérez A., Sánchez-Jiménez F., Vilariño-García T., Sánchez-Margalet V. (2020). Role of Leptin in Inflammation and Vice Versa. Int. J. Mol. Sci..

[14] Laule C., Rahmouni K. (2025). Leptin and Associated Neural Pathways Underlying Obesity-Induced Hypertension. Compr. Physiol..

[15] Casado M.E., Collado-Pérez R., Frago L.M., Barrios V. (2023). Recent Advances in the Knowledge of the Mechanisms of Leptin Physiology and Actions in Neurological and Metabolic Pathologies. Int. J. Mol. Sci..

[16] Wang C., Chang L., Wang J., Xia L., Cao L., Wang W., Xu J., Gao H. (2023). Leptin and risk factors for atherosclerosis: A review. Medicine.

[17] Azar S.T., Zalloua P.A., Zantout M.S., Shahine C.H., Salti I. (2002). Leptin levels in patients with type 1 diabetes receiving intensive insulin therapy compared with those in patients receiving conventional insulin therapy. J. Endocrinol. Investig..

[18] Belin de Chantemèle E.J. (2017). Sex Differences in Leptin Control of Cardiovascular Function in Health and Metabolic Diseases. Adv. Exp. Med. Biol..

[19] Cheng J., Luo Y., Li Y., Zhang F., Zhang X., Zhou X., Ji L. (2022). Sex- and body mass index-specific reference intervals for serum leptin: A population based study in China. Nutr. Metab..

[20] Redondo M.J., Siller A.F., Gu X., Tosur M., Bondy M., Devaraj S., Sisley S. (2020). Sex differences in circulating leptin as a marker of adiposity in obese or overweight adolescents with type 1 diabetes. BMJ Open Diabetes Res. Care.

[21] Isidori A.M., Strollo F., Morè M., Caprio M., Aversa A., Moretti C., Frajese G., Riondino G., Fabbri A. (2000). Leptin and aging: Correlation with endocrine changes in male and female healthy adult populations of different body weights. J. Clin. Endocrinol. Metab..

[22] Lausten-Thomsen U., Christiansen M., Fonvig C.E., Trier C., Pedersen O., Hansen T., Holm J.C. (2015). Reference values for serum total adiponectin in healthy non-obese children and adolescents. Clin. Chim. Acta.

[23] Kuo S.M., Halpern M.M. (2011). Lack of association between body mass index and plasma adiponectin levels in healthy adults. Int. J. Obes..

[24] Cosentino F., Grant P.J., Aboyans V., Bailey C.J., Ceriello A., Delgado V., Federici M., Filippatos G., Grobbee D.E., Hansen T.B. (2020). 2019 ESC Guidelines on diabetes, pre-diabetes, and cardiovascular diseases developed in collaboration with the EASD. Eur. Heart J..

[25] SCORE2-Diabetes Working Group and the ESC Cardiovascular Risk Collaboration (2023). SCORE2-Diabetes: 10-year cardiovascular risk estimation in type 2 diabetes in Europe. Eur. Heart J..

[26] Paliares I.C., Dualib P.M., Torres L.S.N., Aroucha P.M.T., de Almeida-Pititto B., de Sa J.R., Dib S.A. (2024). Evaluation of the Steno Type 1 Risk Engine in predicting cardiovascular events in an ethnic mixed population of type 1 diabetes mellitus and its association with chronic microangiopathy complications. Cardiovasc. Diabetol..

[27] Kaza M., Tsentidis C., Vlachopapadopoulou E., Sakou I.I., Karanasios S., Mastorakos G., Karavanaki K. (2022). The Effect of Metabolic Profile on Leptin, Adiponectin, and hs-CRP in Children and Adolescents with Type 1 Diabetes. Children.

[28] Le Caire T., Palta M. (2015). Longitudinal Analysis of Adiponectin through 20-Year Type 1 Diabetes Duration. J. Diabetes Res..

[29] Zhang M.Y., Dini A.A., Yang X.K., Li L.J., Wu G.C., Leng R.X., Pan H.F., Ye D.Q. (2017). Association between serum/plasma adiponectin levels and immune-mediated diseases: A meta-analysis. Arch. Dermatol. Res..

[30] Frühbeck G., Gómez-Ambrosi J., Muruzábal F.J., Burrell M.A. (2001). The adipocyte: A model for integration of endocrine and metabolic signaling in energy metabolism regulation. Am. J. Physiol. Endocrinol. Metab..

[31] Vilariño-García T., Polonio-González M.L., Pérez-Pérez A., Ribalta J., Arrieta F., Aguilar M., Obaya J.C., Gimeno-Orna J.A., Iglesias P., Navarro J. (2024). Role of Leptin in Obesity, Cardiovascular Disease, and Type 2 Diabetes. Int. J. Mol. Sci..

[32] Poetsch M.S., Strano A., Guan K. (2020). Role of Leptin in Cardiovascular Diseases. Front. Endocrinol..

[33] Katsiki N., Mikhailidis D., Banach M. (2018). Leptin, cardiovascular diseases and type 2 diabetes mellitus. Acta Pharmacol. Sin..

[34] Sáinz N., Barrenetxe J., Moreno-Aliaga M.J., Martínez J.A. (2015). Leptin resistance and diet-induced obesity: Central and peripheral actions of leptin. Metabolism.

[35] Atwa H., Shora H., Elsayed A. (2018). Hormonal, Metabolic and Radiological Markers of Subclinical Atherosclerosis in Egyptian Children with Type 1 Diabetes. Rep. Endocr. Disord..

[36] D’Elia L., Giaquinto A., De Luca F., Strazzullo P., Galletti F. (2020). Relationship between circulating leptin levels and arterial stiffness: A systematic review and meta-analysis of observational studies. High Blood Press. Cardiovasc. Prev..

[37] Sheriff D. (2017). Adiponectin, Diabetes and Coronary Artery Disease. JAMA.

[38] Chausheva G. (2023). Laboratory Cardiovascular Risk Assessment in Individuals with Long-Standing Type 1 Diabetes Mellitus—Adipokines, Osteoprotegerin, Asymmetric Dimethyl-Arginine [Dissertation].

